# When the clock ticks wrong with COVID‐19

**DOI:** 10.1002/ctm2.949

**Published:** 2022-11-17

**Authors:** Silvana Papagerakis, Raed Said, Farinaz Ketabat, Razi Mahmood, Meenakshi Pundir, Liubov Lobanova, Greg Guenther, Giuseppe Pannone, Kerry Lavender, Blake R. McAlpin, Alain Moreau, Xiongbiao Chen, Petros Papagerakis

**Affiliations:** ^1^ Laboratory of Oral, Head and Neck Cancer – Personalized Diagnostics and Therapeutics, College of Medicine University of Saskatchewan Saskatoon Saskatchewan Canada; ^2^ Department of Surgery, College of Medicine University of Saskatchewan Saskatoon Saskatchewan Canada; ^3^ Division of Biomedical Engineering University of Saskatchewan Saskatoon Saskatchewan Canada; ^4^ Department of Biochemistry, Microbiology and Immunology, College of Medicine University of Saskatchewan Saskatoon Saskatchewan Canada; ^5^ Department of Otolaryngology – Head and Neck Surgery, Medical School The University of Michigan Ann Arbor Michigan USA; ^6^ Laboratory of Precision Oral Health and Chronobiology, College of Dentistry University of Saskatchewan Saskatoon Saskatchewan Canada; ^7^ Department of Anatomy, Physiology and Pharmacology, College of Medicine University of Saskatchewan Saskatoon Saskatchewan Canada; ^8^ Anatomic Pathology Unit, Department of Clinic and Experimental Medicine University of Foggia Foggia Italy; ^9^ Laboratories of Neuroimmunology, Department of Symptom Research, Division of Internal Medicine The University of Texas MD Anderson Cancer Center Houston Texas USA; ^10^ Viscogliosi Laboratory in Molecular Genetics of Musculoskeletal Diseases Centre Hospitalier Universitaire (CHU) Sainte‐Justine Research Center Montreal Quebec Canada; ^11^ Department of Stomatology, Faculty of Dentistry and Department of Biochemistry and Molecular Medicine, Faculty of Medicine Université de Montréal Montreal Quebec Canada; ^12^ Department of Mechanical Engineering, School of Engineering University of Saskatchewan Saskatoon Saskatchewan Canada

**Keywords:** circadian clock, clinical outcomes, COVID‐19, epigenetics, microRNAs, oral and systemic precision health, personalized medicine, SARS‐CoV‐2 infection

## Abstract

Severe acute respiratory syndrome coronavirus 2 (SARS‐CoV‐2) is a member of the coronavirus family that causes the novel coronavirus disease first diagnosed in 2019 (COVID‐19). Although many studies have been carried out in recent months to determine why the disease clinical presentations and outcomes can vary significantly from asymptomatic to severe or lethal, the underlying mechanisms are not fully understood. It is likely that unique individual characteristics can strongly influence the broad disease variability; thus, tailored diagnostic and therapeutic approaches are needed to improve clinical outcomes. The circadian clock is a critical regulatory mechanism orchestrating major physiological and pathological processes. It is generally accepted that more than half of the cell‐specific genes in any given organ are under circadian control. Although it is known that a specific role of the circadian clock is to coordinate the immune system's steady‐state function and response to infectious threats, the links between the circadian clock and SARS‐CoV‐2 infection are only now emerging. How inter‐individual variability of the circadian profile and its dysregulation may play a role in the differences noted in the COVID‐19‐related disease presentations, and outcome remains largely underinvestigated. This review summarizes the current evidence on the potential links between circadian clock dysregulation and SARS‐CoV‐2 infection susceptibility, disease presentation and progression, and clinical outcomes. Further research in this area may contribute towards novel circadian‐centred prognostic, diagnostic and therapeutic approaches for COVID‐19 in the era of precision health.

## Graphical abstract

SARS‐CoV‐2 entry happens through the oral and nasal cavities. Oral and/or nasal preexisting dysbiosis may affect COVID‐19 susceptibility, severity and outcomes. Bidirectional interactions between gut microbiota and the oral/nasal/lung microbiota may be involved in regulating the immune responses to SARS‐CoV‐2, which may involve the circadian clock

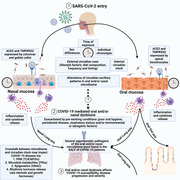

## 1 INTRODUCTION: GENERAL ASPECTS ON CIRCADIAN CLOCK AND COVID‐19

The novel coronavirus disease 2019 (COVID‐19) pandemic, caused by severe acute respiratory syndrome coronavirus 2 (SARS‐CoV‐2), is the largest global public health crisis since the outbreak of influenza in 1918.^1^ At the time of writing, nearly 520 million COVID‐19 cases have been confirmed globally, and the COVID‐19 death toll is approximately 6.2 million deaths worldwide.^2^ Despite the growing rate of SARS‐CoV‐2 infections, with poor outcomes especially in individuals with comorbidities, the clinical patterns of COVID‐19 disease progression remain unpredictable.^3^, ^4^

The circadian clock plays an essential role in regulating daily physiological processes and coordinating the innate and adaptive functioning of the immune system.^5^ Recent evidence suggests that a healthy circadian rhythm corroborated with a specific timing of infection may contribute to decreased viral replication and dissemination, highlighting the clinical relevance of circadian biology in emerging infectious diseases such as SARS‐CoV‐2 infection.^5^, ^6^ Here, we have summarized the most representative information to increase our understanding of the relationship between circadian rhythmicity and SARS‐CoV‐2 infection. After a brief introduction on COVID‐19 symptoms, associated clinical outcomes and their relationship to the circadian clock, we have taken a deeper look at the potential mechanistic links between the circadian clock, immune system and viral infections with an emphasis on COVID‐19 infection. We have also summarized the circadian clock‐related determinants of COVID‐19 infection, including demographic characteristics, genetic profile, immune aspects, comorbidities, lifestyle and environmental factors. Lastly, we have also considered studies investigating the potential that melatonin and lifestyle changes (such as dietary aspects) may have in manipulating the circadian clock machinery to improve outcomes and recovery from COVID‐19 disease. For this review, we have undertaken a comprehensive search through MEDLINE, EMBASE, Scopus, LitCovid and Web of Science using the following keywords: circadian clock disruption, COVID‐19, SARS‐2, angiotensin‐converting enzyme (ACE)/angiotensin‐converting enzyme 2 (ACE2), Clock Genes, Epithelium, Immunity, Infection, Chronotherapy, Sex differences, Microbiome and Melatonin.

## 2 COVID‐19 CHALLENGES AND BROAD DISEASE VARIABILITY – CIRCADIAN CLOCK DISRUPTION AS POTENTIAL MEDIATOR AND REGULATOR OF COVID‐19 UNPREDICTABLE OUTCOMES

COVID‐19 infection caused by SARS‐CoV‐2 shares some pathological features with other coronavirus‐related infections such as severe acute respiratory syndrome (SARS) and Middle East respiratory syndrome (reviewed by Zhu et al.^7^). Symptoms reported in patients presenting with COVID‐19 infection are extremely varied ranging from fever, fatigue, cough, sputum production, haemoptysis, breathlessness, headache, myalgia, diarrhoea, loss of taste and/or smell, chilblain‐like skin lesions (referred to as ‘COVID toe’), and so on.^8^, ^9^, ^10^, ^11^, ^12^ In addition, central nervous system (CNS)‐related symptoms, such as fatigue, sleep disorders and cognitive impairment, have been noted in both mild and severe COVID‐19 infections.^13^, ^14^, ^15^ COVID‐19 infection can result in a wide range of complications, including acute respiratory distress syndrome (ARDS), numerous severe cardiovascular complications, including a distinctive coagulopathy (COVID‐19‐associated coagulopathy), lymphopenia, acute kidney injury and multi‐organ dysfunction or failure (liver, kidney and heart) that lead to death in a subset of patients.^8^, ^9^, ^16^, ^17^ Importantly, of increasing concern is the phenomenon of ‘long‐COVID’ or ‘post‐COVID syndrome’ in which symptoms persist for months after recovery from active COVID‐19 infection.^18^ Researchers have suggested that long‐COVID may be mediated in part by neuroinflammation, but additional research is required to understand long‐COVID prevalence and risk factors.^18^, ^19^

Echoing the general consensus, a China‐based study indicated that COVID‐19‐positive patients, as determined by viral nucleic acid or antibody testing, can be stratified into either symptomatic (those complaining of classic symptoms like fever, dry cough and fatigue at their initial presentation) or asymptomatic (without symptoms at their initial screening) groups. However, a portion of the initially asymptomatic patients can be later re‐classified into the symptomatic group if their clinical outcomes worsen or they develop additional symptoms, likely because these patients were pre‐symptomatic at the time of the initial screening (incubation period).^20^ It is thus generally accepted that only those individuals who remain asymptomatic and never become ill are the ‘true’ asymptomatic cases.^20^ Considerable efforts, with most representative evidence summarized in this review, have been made towards identifying prognostic biomarkers and predisposing risk factors for the prediction of the disease outcome. However, disease progression monitoring tools that are tailored to the wide range of clinical presentations by considering the individual comprehensive health profile are yet to be validated.

Cumulative evidence indicates that mildly symptomatic COVID‐19‐infected patients who received appropriate medical care underwent a shorter recovery period than those severely ill with COVID‐19. Older age and comorbidities such as cardiovascular disease, diabetes mellitus (DM), obesity and hypertension seem to contribute to increased disease severity, increased complications, worsened outcome and a higher mortality rate.^17^, ^21^

A China‐based study of 221 COVID‐19 patients who fully recovered estimated that the recovery time in those with mild versus severe disease was 10.63 ± 1.93 versus 18.70 ± 2.50 days, respectively. Recovery time was calculated based on the number of days from the first positive to the first negative nucleic acid test (NAT). Of note, COVID‐19 infection status was diagnosed using NAT in specimens collected from the throat and/or nose of each patient.^22^

Worldwide, the overall case fatality rate (CFR) in COVID‐19‐infected patients has been reported to be between 2% and 3%.^23^ Among 38 countries reporting sex disaggregated data, 37 reported higher COVID‐19 mortality rates among males compared to females.^24^ It is important to note that the CFR might be affected by additional factors besides sex, including age, race, comorbidities, socioeconomic status, access and quality of healthcare, type of treatment, timing of interventions and more.^24^, ^25^, ^26^, ^27^ Studies are still needed to fully understand the impact of additional variables such as environmental, genetic, lifestyle factors and others that may influence COVID‐19 outcomes to develop reliable prognostic biomarkers. Therefore, this review aims to explore the relationship between circadian rhythmicity, viral susceptibility and the immune response to improve our understanding of the impact that circadian rhythmicity has on COVID‐19 disease severity and outcomes.

## 3 LINKS BETWEEN CIRCADIAN CLOCK AND COVID‐19

The circadian rhythm is an adaptation to the 24‐h light/dark cycle; almost all living organisms have developed circadian clock rhythms which usually have 24‐h oscillations to help adapt their functions and physiology to environmental changes.^28^ The mammalian circadian clock comprises the central clock and peripheral clocks. The central clock is located in the suprachiasmatic nuclei (SCN) of the hypothalamus, whereas peripheral clocks are found in peripheral tissues and organs. The central and peripheral clocks are synchronized through hormonal and neural pathways; however, peripheral clocks may oscillate self‐sustained for over 20 cycles during a 24‐h period in isolation.^29^, ^30^, ^31^, ^32^, ^33^ At the molecular level, clocks are regulated by perpetual transcriptional/translational feedback loops.^34^ Aryl hydrocarbon receptor nuclear translocator‐like protein 1 (also called brain and muscle ARNT‐like 1 [BMAL]) and Circadian Locomotor Output Cycles Kaput (CLOCK) are two clock proteins that form a heterodimer in the morning and bind to the E‐box promoter triggering the expression of Period (PER) and Cryptochrome (*CRY*) genes.^35^ Accumulation of PER and CRY and their heterodimerization in the evening prevent the activity of BMAL1 and CLOCK. Therefore, a rhythmic oscillation occurs within a period of about 24 h.^36^

The circadian clock is involved in regulating many physiological processes in the body.^37^ A healthy individual has a synchronized circadian clock system working in harmony between the central and peripheral clocks. The peripheral clocks are also synchronized independently of the central clock in different organs.^38^ Loss of synchronization between the central and peripheral clock may lead to the onset and progression of various diseases such as hypertension and cardiovascular disease.^38^, ^39^ Scheer et al. investigated the relationship between sleep/wake and feeding/fasting cycles (behavioural cycles) with endogenous circadian cycles as predictors of diabetes, obesity and cardiovascular risk. This clinical study was conducted on 10 volunteers (5 males, 5 females; aged 19–41‐year old, mean age of 25.5 years) by scheduling 28‐h daily cycles instead of 24 h. This circadian misalignment led to decreased leptin, increased glucose albeit with increased insulin, increased mean arterial pressure, a complete reversal of the daily cortisol rhythm, and reduced sleep efficiency, highlighting the broad impact that even a relatively short circadian disruption may have on critical biological processes in the human body.^40^ Considering the relationship between these comorbidities and COVID‐19 disease severity, the influence that circadian disruption has on COVID‐19 outcomes should be further explored.

In addition to the circadian regulation of physiological functions, Mure et al. recently reported that 82.2% of the genes encoding proteins that are targets of therapeutic drugs used in the clinical practice have a rhythmic expression in at least one tissue.^41^ This provides an opportunity for the optimization of drug delivery schedules based on circadian profiles of targets, transporters, and respective metabolizing enzymes for each drug. Therefore, knowledge of circadian clock functioning may aid in disease prevention, including COVID‐19, as well as increasing the effectiveness of treatments and reducing adverse effects.

We later summarized the current state of knowledge on how the circadian clock can regulate different physiological systems and possible connections with COVID‐19 infection's progression and outcomes.

### 3.1 Circadian clock – a determinant of anti‐viral immune defence

It has become clear that both innate and adaptive immune systems are modulated by the circadian clock at almost every level starting from mobilization, trafficking and chemotaxis of leukocytes to release of cytokines and T‐cell differentiation.^42^ For example, *BMAL1* and *REV‐ERBα* clock genes are among the circadian system's transcriptional factors responsible for regulating immune cell trafficking.^43^

#### 3.1.1 Innate immunity and the circadian clock

Macrophages, monocytes, neutrophils, eosinophils, mast cells and natural killer (NK) cells are different types of innate immune cells that function according to their intrinsic clocks. For instance, neutrophil maturation and antimicrobial activity occur rhythmically.^44^, ^45^ It was suggested that each immune cell's physiology is mediated by some specific clock genes.^46^ Splenic NK cells need *PER1* for efficient functioning.^46^, ^47^ Logan et al. reported alteration in the rhythmicity of cytokine and cytolytic factors in *Per1* ^−/−^ mice.^47^ Sato et al. showed that *REV‐ERBα* controls the inflammatory functions of macrophages (including adhesion, migration and integrin activation) by the regulation of CC‐chemokine ligand 2 expression.^46^, ^48^

**FIGURE 1:**
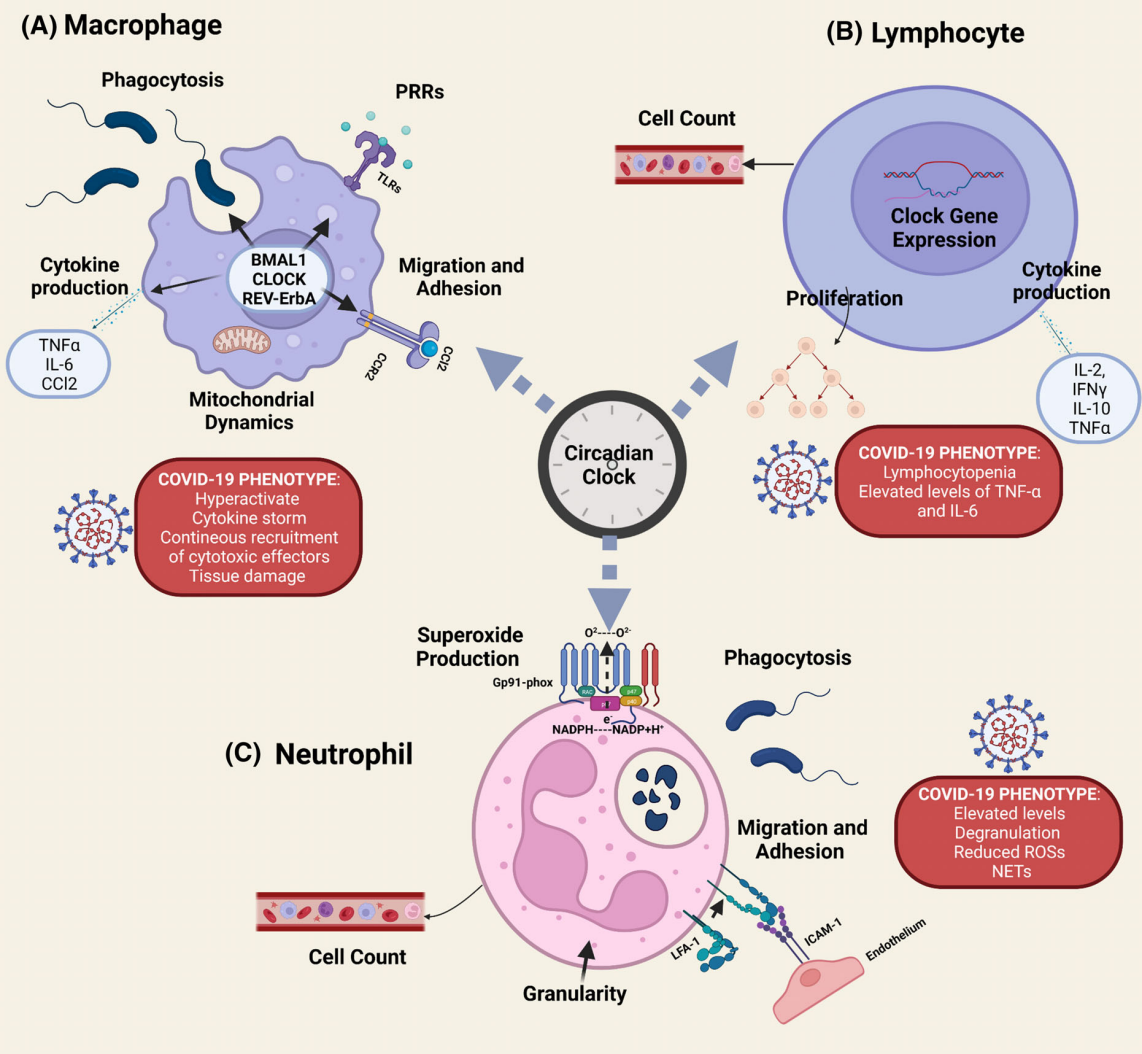
Circadian clock – a determinant of immune defence and its links to coronavirus disease 2019 (COVID‐19). Both innate and adaptive immune systems are modulated by the circadian clock at almost every level. This is a schematic representation of the known aspects of monocyte and macrophage (A), lymphocyte (B) and neutrophil (C) that are regulated by the circadian clock. (A) Macrophages and monocytes have been the most studied cell types in the context of circadian rhythms. Macrophages exhibit a high amplitude of clock gene expression that appear to modulate several macrophage functions, including phagocytosis, the expression of pattern‐recognition receptors (PRRs), recruitment to tissues, mitochondrial dynamics and cytokines in response to a challenge^48,^ ^237^, ^238^, ^239^, ^240^, ^241^ suggesting that circadian misalignment is involved in the acquiring of the hyperactivated pulmonary macrophage phenotype observed in COVID‐19 patients resulting in a damaging loop of pro‐inflammatory cytokine release and recruitment of other cytotoxic effector cells thereby exacerbating tissue damage.^242^ (B) Both B and T cells in mouse lymph nodes express several clock in a rhythmic manner.^243^ In human blood, T‐ and B‐cell numbers varied throughout the day and were found to be higher at night and then declined in the morning.^244^ A circadian rhythm of cytokine production (IL‐2, IFN‐γ, IL‐10 and TNF‐α) was also observed after the TCR stimulation of T helper cells in vitro.^245^ Both CD4+ and CD8+ T‐cell proliferation after antigenic stimulation showed circadian rhythms with stronger proliferation during the late subjective day and during the subjective night.^243^ The circadian control of lymphocyte proliferation and cytokine production may be involved in the lymphocytopenia observed in patients with COVID‐19 where there is a marked reduction in the CD4+ T lymphocyte number and elevated levels of TNF‐α and IL‐6 that correlate with the severity of COVID‐19 disease.^246^ (C) Peripheral neutrophils also express components of the molecular clock, and endotoxin administration was found to downregulate clock genes (Clock, Per3, Cry1‐2, Rev‐erb and Rora) expression in human neutrophils.^247^ Daily variations in neutrophil count and functions, such as superoxide production (i.e. rhythmic Gp91phox expression), phagocytosis and expression of cell adhesion molecules (e.g. l‐selectin, ICAM1 and LFA‐1), have been described in several studies, suggesting a significant impact of the circadian clock on neutrophil activity.^248^ The previous description suggests that the circadian disruption may exacerbate the altered neutrophil abundance, phenotype and functionality observed in COVID‐19 patients that include elevated neutrophil levels, increased degranulation reduced reactive oxygen species release and heightened capacity for neutrophil associated extracellular trap formation^249^). Some disagreement regarding the role of circadian clock in immunity are raised^250^ and more studies are needed. *Source*: BioRender.com

Narasimamurthy et al. studied *Cry1* and *Cry2* double knockout mice to measure the expression of inflammatory mediators in the hypothalamus. The expression of interleukin 6 (*IL‐6*), C‐X‐C Motif Chemokine Ligand 1 (*Cxcl1*), tumour necrosis factor‐alpha (*TNF‐α*) and inducible nitric oxide synthase (iNOS) was significantly elevated in *Cry1* and *Cry2* double knockout mice compared to the wild‐type mice.^49^ The authors found that in the absence of CRY proteins, mammalian cells express inflammatory cytokines constantly, which causes a condition called ‘metaflammation’ or ‘low‐grade chronic inflammatory status’.^49^ Oishi et al. have shown that the *Bmal1* gene is expressed in macrophages and can regulate the timing of genes expressed in response to inflammatory activation, partially via the regulation of the transcription of *REV‐ERBα/β* and enhancer RNAs (eRNA) in macrophages using *Bmal1^−/−^* cells and mouse models.^50^ It is important to note that influence of the circadian clock on inflammatory mediators is bidirectional: Although circadian disruption is known to promote chronic inflammatory and metabolic diseases, inflammation can also directly affect the circadian clock.^51^ In fact, the pro‐inflammatory cytokine TNF‐α has been shown to suppress the expression of clock‐controlled genes.^52^ The impact of severe inflammatory response, called the cytokine storm syndrome (CSS), on COVID symptoms and fatality is well documented and has been proposed to be mediated by NOD‐like receptor 3 (NLRP3) inflammasome activation. Therefore, the bidirectional relationship between inflammatory mediators and circadian rhythmicity should be further explored in the context of COVID‐19. Taken together, the innate immune system and circadian clock maintain a strong reciprocal relationship (Figure 1).

#### 3.1.2 Adaptive immunity and circadian clock

Despite the fact that the adaptive immune response develops over a longer period that may extend to weeks or months, many of its aspects are under circadian control. Adaptive immune cells, including T and B cells, show circadian oscillation.^46^ In fact, Silver et al. have demonstrated that even dendritic cells, a heterogenous family of immune cells positioned at the interface between innate and adaptive immunity, show oscillations in the core clock components, including PER1, PER2, BMAL1, REV‐ERBα and a clock‐controlled gene, D‐Box Binding PAR BZIP Transcription Factor (*Dbp*).^46^, ^53^

In summary, both innate and adaptive immune systems are modulated by the circadian clock at almost every level, and these interactions may be crucial in COVID‐19 outcomes heterogeneity (Figure 1).

### 3.2 Circadian clock and susceptibility to viral infections

#### 3.2.1 Melatonin as a chronomodulator of anti‐viral immunity

Humans seem more prone to infections during the rest period at night and less susceptible during the day. This could be explained by the circadian behaviour of lymphocyte migration into lymph nodes at night onset, followed by their return to tissues in the morning.^54^ A direct implication of the circadian clock mechanism in viral infections has been suggested by the therapeutic benefit of melatonin.^55^ Melatonin is a tryptophan‐derived indole that is mainly secreted by the pineal gland during nighttime and plays a major role in circadian entrainment of several peripheral tissues with special emphasis on immune cells.^56^ It has been suggested that the regulatory effect of melatonin on the circadian regulation of immune responses is largely mediated via the melatonergic mitochondrial pathways.^57^ Briefly, melatonin acts to supress and reset immune cell activity during night/rest time by an induction of the core circadian gene *Bmal1* which, in turn, disinhibits the mitochondrial pyruvate decarboxylase (PDC)‐acetyl CoA pathway leading to increased oxidative phosphorylation and tricyclic acid circle cycle activity in immune cells.^58^ This melatonin‐induced shift towards aerobic oxidation instead of glycolytic metabolism during rest periods results in more quiescent immune cells with dampened inflammatory activity in order to prime the immune cells for daytime activation.^58^ In addition to the BMAL1‐mediated regulation of mitochondrial metabolism, melatonin acts as a chronomodulator of immunity via several other molecular pathways. Indeed, it was found that melatonin exerts its anti‐inflammatory effects via the inhibition of the pro‐inflammatory nuclear factor kappa‐light‐chain‐enhancer of activated B cells (NF‐κB) pathway in both T cells and lung tissues.^59^ The anti‐inflammatory and anti‐oxidative effects of melatonin may also be mediated via the activation of mitochondrial Sirtuin‐1 (SIRT1), and inhibition of toll‐like receptor 4 (TLR4) and NLRP3 signalling resulting in a reduced shift of macrophages towards a pro‐inflammatory state with subsequent marked reduction of pro‐inflammatory cytokines, such as TNF‐α, IL‐1β and IL‐6, and IL‐8, and an elevation in the levels of the anti‐inflammatory cytokine IL‐10.^59^, ^60^, ^61^, ^62^ In fact, research has shown that the use of melatonin as a medicinal supplement in COVID‐19 patients robustly reduced markers of oxidative stress (MDA, NO and SOD) and inflammasome activation as identified by the expression of *CASP1* and *ASC* which contribute to NLRP3‐associated inflammasome activation. SIRT1 activation by melatonin has also been shown to reduce neuroinflammation and oxidative stress in the brain.^63^, ^64^ Finally, it has been reported that melatonin facilitates the proliferation and maturation of both the lymphoid and myeloid immune cell lineages in the bone marrow and that melatonin enhances the antigen presentation capacity of macrophages.^62^, ^65^, ^66^, ^67^ Taken together, it is clear that melatonin acts as potent circadian regulator of immunity with general anti‐inflammatory, anti‐oxidative and cytoprotective effects. These effects were observed in several viral infection and non‐viral respiratory distress and lung injury models which strongly suggest that melatonin may be utilized as a therapeutic adjuvant in COVID‐19 infection, similar to what has been observed in other viral infections as summarized in the following.^60^, ^68^

#### 3.2.2 Melatonin and its potential benefits against viral infections

##### Influenza A, particularly the hemagglutinin type 1 and neuraminidase type 1 (H1N1) strain

H1N1 viral infection results in an uncontrolled inflammatory response in the lungs, particularly the production of TNF‐α and interferon‐gamma (IFN‐γ) by human influenza A virus (IAV)‐specific CD8^+^ T cells.^69^ Studies have reported a strong therapeutic benefit of melatonin in infections with IAV, due to its anti‐inflammatory, anti‐oxidative and immuno modulatory effects.^59^, ^69^ Treatment with melatonin resulted in a significantly increased production of IL‐10 and transforming growth factor‐beta and inhibited the production of TNF‐α in CD8^+^ T cells of BALB/c mice infected with influenza H1N1.^69^

##### Ebola virus

The Ebola virus and its resulting infection led to impairments of the immune system, increased blood coagulation and induction of an inflammatory response that caused massive oxidative damages resulting in cellular and organ failure. Reiter et al. found that melatonin was beneficial in treating Ebola virus‐infected patients by affecting thrombin formation and platelet physiology, providing additional supporting evidence for melatonin's anti‐viral benefit.^70^

##### Venezuelan equine encephalitis (VEE)

It has been shown that Venezuelan equine encephalitis (VEE) may cause an early production of IFN‐α and ‐β followed by later production of virus‐specific neutralizing antibodies.^71^ Reports indicated that the levels of TNF‐α and IL‐1β were significantly increased in the brains of male albino mice infected with the VEE virus.^72^ The study reported a decrease in TNF‐α synthesis after the treatment of VEE‐infected mice with melatonin, suggesting a protective effect of melatonin against the VEE virus. Melatonin treatment resulted in a significant increase of IL‐1β only in the infected mice, whereas in the non‐infected control mice the levels remained unaffected.^72^

##### Respiratory syncytial virus (RSV)

The mechanisms underlying respiratory syncytial virus (RSV)‐induced disease remain largely unknown. Still, it was suggested that the host's oxidative stress response may play a significant role in RSV‐induced lung infection.^73^ Huang et al. have studied the relationship between the pathogenesis of RSV‐induced lung inflammation and oxidative stress, and the effects of exogenous melatonin in mice with RSV‐induced oxidative pulmonary injury.^73^ In this study, the inflammatory and oxidative status in RSV‐infected female BALB/c mouse lungs was significantly altered as indicated by the increased expression of TNF‐α and nitric oxide, respectively. In RSV‐infected mice, exogenous melatonin ameliorated lung injury by reducing the production of pro‐inflammatory cytokines and inhibiting the oxidative stress response.^73^

Considering the beneficial anti‐inflammatory, anti‐oxidative and cytoprotective effects of melatonin in regards to the aforementioned viral inflections, the potential links between COVID‐19 disease severity and melatonin's circadian regulation of immunity needs to be explored.

#### 3.2.3 COVID‐19 and melatonin metabolism

**FIGURE 2:**
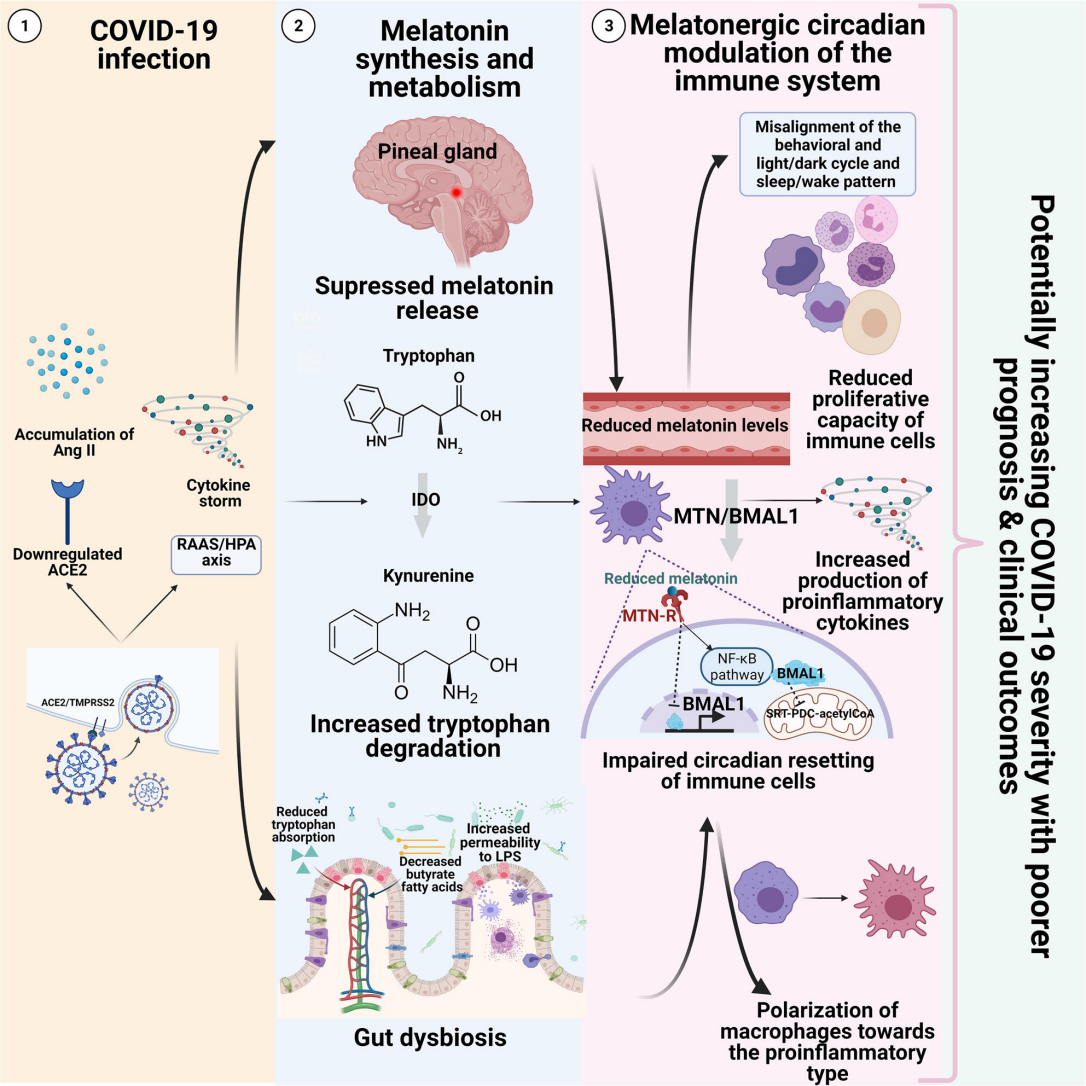
Reciprocal links between coronavirus disease 2019 (COVID‐19) and different melatonergic pathways suggestive of melatonin as a viable therapeutic target and chronomodulator of COVID‐19 infection. (1) Severe acute respiratory syndrome coronavirus 2 (SARS‐CoV‐2) entry into host cells results in a pro‐inflammatory cytokine storm that is further exacerbated by the constitutive activation of the renin–angiotensin activating system along with the hypothalamic pituitary adrenal axis due to angiotensin II (Ang II) accumulation and the loss of surface angiotensin converting enzyme. (2) The pro‐inflammatory cytokines may suppress melatonin production in the pineal gland via the immune pineal axis and increased cytokine levels lead to the activation of the indoleamine 2,3‐dioxygenase (IDO) and tryptophan 2,3‐dioxygenase (TDO) enzymes. IDO/TDO activation promotes the degradation of tryptophan into kynurenine and reduces the rate of tryptophan conversion into melatonin. Melatonin metabolism will be also affected by cytokine‐mediated gut dysbiosis and impaired gut permeability, which may lead to reduced uptake of tryptophan and the microbiome‐derived short chain fatty acid butyrate accompanied by increased permeability to lipopolysaccharide (LPS) which can induce further cytokine production. (3) This results in a marked decrease in melatonin levels and the corresponding loss of its anti‐inflammatory, anti‐oxidative, immune enhancing and cytoprotective effects, which are largely mediated via the induction of Bmal1 disinhibition of the mitochondrial pyruvate decarboxylase–acetyl CoA and inhibition of the pro‐inflammatory NF‐κB pathway. Moreover, the altered melatonin levels will disrupt the sleep/wake cycles of COVID‐19 patients which will further increase their susceptibility. All the previously mentioned developments are expected to increase the severity of inflammation and worsen disease progression. ACE2, angiotensin converting enzyme 2; BMAL1, brain and muscle ARNT‐like 1; HPA, hypothalamic pituitary adrenal; MTN, melatonin; MTN‐R, melatonin receptors; NF‐κB, nuclear factor kappa‐light‐chain‐enhancer of activated B cells; PDC, pyruvate decarboxylase; RAAS, renin–angiotensin aldosterone system; Sirt, Sirtuin. *Source*: BioRender.com

The cytokine storm observed in COVID‐19 patients has the potential to greatly affect melatonin synthesis and metabolism.^58^ For instance, pro‐inflammatory cytokines may suppress melatonin production in the pineal gland via the immune pineal axis, and increased cytokine levels lead to the activation of the indoleamine 2,3‐dioxygenase (IDO) enzyme.^74^ IDO activation promotes the degradation of tryptophan into kynurenine and reduces the rate of tryptophan conversion into serotonin and subsequently melatonin by arylalkylamine *N*‐acetyltransferase and acetylserotonin methyltransferase.^75^, ^76^ In turn, kynurenine metabolites activate BMAL1‐controlled aryl hydrocarbon receptor (AhR) that modulates the host antiviral response causing additional cytokine production and increased severity of the inflammatory response.^77^, ^78^ In addition, the cytokine‐mediated gut dysbiosis and impaired gut permeability may lead to a reduced intake of the microbiome‐derived short chain fatty acid butyrate and increased permeability to lipopolysaccharide (LPS).^58^, ^79^, ^80^ Reduced butyrate leads to a reduced disinhibition of PDC‐acetyl CoA pathway, whereas LPS infiltration can induce further cytokine production.^80^, ^81^ Furthermore, the loss of ACE2 in the gut due to COVID‐19 infection may lead to reduced tryptophan absorption.^82^ Collectively, all the above‐mentioned pathways can result in a marked decrease in melatonin levels and the resulting loss of its anti‐inflammatory, anti‐oxidative, immune enhancing and cytoprotective effect in COVID‐19 patients to further exacerbate the severity of inflammation and worsen disease progression. The plausible interactions between COVID‐19 and melatonergic circadian pathways are illustrated in Figure 2.

#### 3.2.4 Roles of clock genes in viral infections

In addition to melatonin's therapeutic benefits, recent studies have started to unravel exciting facts about the relationship between circadian clock genes and viruses.^83^

##### Herpes and influenza A

Edgar et al. were able to prove a clear link between the circadian clock and herpes and IAVs, as well as the relevance of the time of infection.^6^ In this study, C57BL/6J mice were infected with a recombinant luciferase‐expressing virus, murine herpesvirus 4 (*M3:luc* MuHV‐4), at two different times of the day. A 10‐fold increase in viral replication was observed in mice infected at the onset of their resting phase compared to mice that were infected just prior to their active phase. When the experiment was repeated with *Bmal1* knock‐out mice, the results indicated a threefold increase in viral replication at either time of day in mice lacking *Bmal1* compared to wild‐type mice. The authors also infected wild‐type and *Bmal1* ^−^ *^/^* ^−^ NIH 3T3 cells with PB2::Gaussia luciferase IAV and found that the loss of *Bmal1* upregulated IAV protein expression and increased its replication.^6^ In addition, another study determined that *BMAL1* mRNA expression is lower in winter than in summer, most likely due to shorter days (less day light exposure), which could at least partially explain the higher rate of influenza infections and other respiratory viral epidemics during winter.^84^

##### Sendai virus (SeV)

Sendai virus (SeV) is a murine parainfluenza virus type 1 that can cause necrosis and inflammation in the respiratory tract.^85^ Ehlers et al. found that the deletion or environmental disruption of core clock gene *Bmal1* (induced by chronic jet lag) could aggravate the acute bronchiolitis caused by SeV but also by the IAV in C57BL6/J mouse models.^86^

##### Respiratory syncytial virus (RSV)

Human epithelial cells of the lower respiratory tract and macrophages produce many chemokines and cytokines in response to RSV.^87^ These include IL‐8, IFN‐γ‐induced protein 10, monocyte chemoattractant protein 1, macrophage inflammatory protein 1 alpha and beta, regulated on activation, normal T expressed and secreted (RANTES, also called CCL‐5), IL‐6, TNF‐α, IL‐1α/β and IFN‐α/β.^87^ Ehlers et al. analysed respiratory tract expression of clock genes (nasal rinse samples) in infants who participated in the RSV Bronchiolitis in Early Life studies.^86^, ^88^ These studies also compared their results with samples taken from healthy adults and paediatric subjects and found that *BMAL1* expression was downregulated compared to healthy controls.^86^ Majumdar et al. infected wild‐type and *Bmal1^−/−^* mice with RSV and monitored body masses and RSV levels in the lung of mice. The *Bmal1^−/−^* mice had more severe loss of body mass than their wild‐type counterparts that never recovered and higher levels of RSV in the lung than wild‐type mice.^89^

##### Herpes simplex virus (HSV)

It is thought that herpes simplex virus‐1 (HSV‐1) viral transactivator–infected cell polypeptide 0 is directly linked with BMAL1, and that the viral transcription is under BMAL1/CLOCK complex control.^6^ However, Matsuzawa et al. suggested that the expression of poliovirus receptor–related 1 (HSV‐2 receptor Nectin‐1) is directly coordinated by the *CLOCK* gene.^90^, ^91^ Kalamvoki and Roizman reported that the *CLOCK* gene could facilitate HSV replication given that the expression of viral genes was suppressed by *CLOCK* gene silencing, which highlights the role of *CLOCK* as a component of the viral transcriptional machinery.^92^, ^93^

##### Hepatitis C virus (HCV)

Benegiamo et al. evaluated the relationship between core clock genes and hepatitis C virus (HCV)‐related viral infection in two in vitro models, the human hepatoma Huh‐7 and the non‐human primate OR6 cell lines and found that *PER2* overexpression decreased HCV RNA replication, suggesting that *PER2* can act as an inhibiting factor of viral replication.^94^

Taken together, these studies highlight the influence that circadian disruption has on disease severity in response to viral infections. Considering the established links that suggest worsened viral infection prognosis in the face of circadian disruption, the relationship between circadian rhythmicity and COVID‐19 infection should be further explored.

### 3.3 Circadian clock and COVID‐19 mechanism of disease

#### 3.3.1 SARS‐CoV‐2 receptors and renin–angiotensin–aldosterone system (RAAS)

SARS‐CoV‐2 infects host cells by binding to the membrane‐bound form of ACE2, which has been previously shown to be regulated by circadian clock gene expression.^95^ ACE2 plays the central role in the interaction between SARS‐CoV‐2 and human cells; however, some other molecules, including transmembrane protease serine 2 (TMPRSS2), extracellular matrix metalloproteinase inducer (CD147), sialic acid receptor and cathepsin L also play an important part in the infectious process.^95^, ^96^, ^97^ Several reports have shown that blocking or reducing ACE2 expression may inhibit cellular entry of SARS‐CoV‐2 into ACE2‐expressing tissues (lung, heart, brain, kidney, gut, nasal cavity olfactory epithelium and mucosa of the oral cavity).^96^, ^98^, ^99^, ^100^, ^101^ ACE2 is one of the critical enzymatic components of the renin–angiotensin–aldosterone system (RAAS), a cascade of vasoactive peptides responsible for regulating critical physiological processes in humans.^102^ This cascade starts with the secretion of a renal hormone, renin. Renin cleaves angiotensinogen into angiotensin I (Ang I), which is converted to angiotensin II (Ang II) by the ACE. Ang II attaches to the Ang II type 1 (AT1) receptor, causing vasoconstriction that initiates the inflammatory response.^103^, ^104^ Then, ACE2 degrades Ang II to Ang‐(1‐7) and prevents the elevation of blood pressure via vasodilation.^104^ In vitro studies have shown that the initial attachment of SARS‐CoV‐2 spike protein to ACE2 is followed by the downregulation of the expression levels of ACE2 on cell surfaces, which may lead to the accumulation of Ang II and activation of local RAAS that might result in lung injury in humans.^102^ These sequence of events has been confirmed by several studies which showed that the administration of recombinant ACE2 may reverse the accumulation of Ang II, inhibiting the adverse effects of SARS‐CoV‐2‐induced lung injury. Similar results have been shown in the context of other viral infections besides SARS‐CoV‐2, such as highly pathogenic avian influenza virus (H5N1) and RSV.^105^, ^106^ Furthermore, it is important to note that clinical studies have reported that the most severe COVID‐19 complications occurred in elderly patients with cardiovascular diseases.^107^ Routine treatment with RAAS inhibitors (ACE inhibitors [ACEI] and angiotensin receptor blockers [ARBs]) can lead to increased synthesis of ACE2 in those patients, increasing their susceptibility to COVID‐19 infection.^108^ However, a recent cohort study did not find an increased risk of COVID‐19 infection, hospitalization and clinical outcomes while comparing ACEI and ARBs to calcium channel blockers and thiazide‐like diuretics.^109^ Finally, a comprehensive literature review by Wang et al., 2020 suggested that the continuation of ACEI and ARBs treatment may be potentially beneficial to COVID‐19 patients.^110^

**FIGURE 3:**
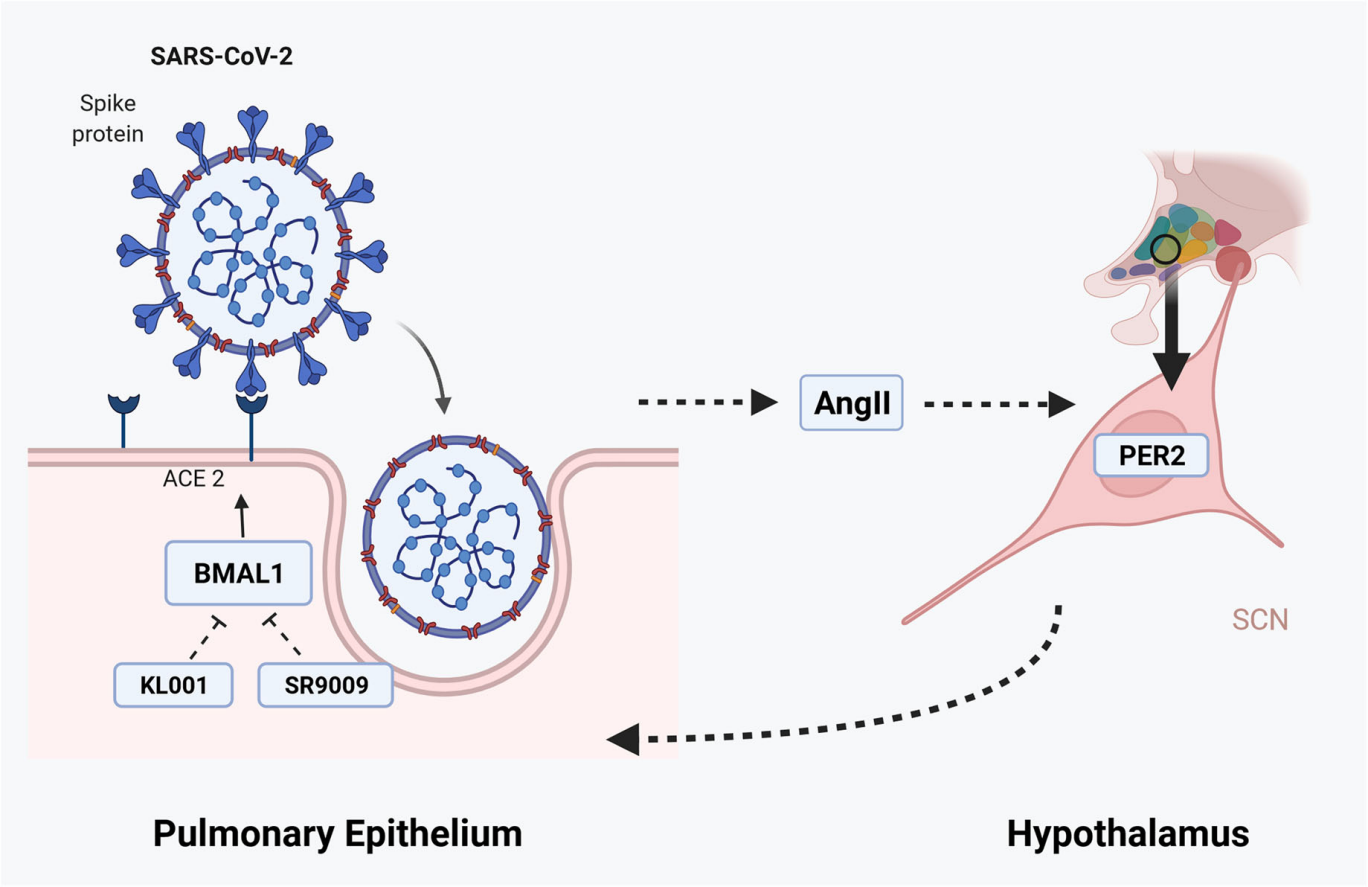
Potential links between angiotensin‐converting enzyme (ACE)/angiotensin‐converting enzyme 2 (ACE2) expression in epithelial cells and circadian clock gene expression in the context of coronavirus disease 2019 (COVID‐19). Targeting the circadian regulator Bmal1 by genetic silencing or by treating lung epithelial cells with the REV‐ERB agonist SR9009 or Cry stabilizer KL001 reduces ACE2 expression and inhibits severe acute respiratory syndrome coronavirus 2 (SARS‐CoV‐2) entry and replication.^251^ Binding of the surface S glycoprotein of SARS‐CoV‐2 to ACE2 may directly compete with the processing of the pro‐inflammatory angiotensin II (Ang II) to the anti‐inflammatory Ang (1–7). Ang II is known to affect the central clock system in the suprachiasmatic nuclei (SCN) through its effect on Per2 levels which may in turn affects the expression of BMAL1 in peripheral tissues, including the pulmonary epithelium.^252^ *Source*: BioRender.com

Studies have indicated that a reciprocal link exists between, ACE2, RAAS and the circadian system,^111^ suggesting that pre‐existing dysregulation of the circadian clock may result in undesirable COVID‐19 outcomes. In fact, both genetic silencing of *Bmal1* and treatment with a REV‐ERB agonist in lung epithelial cells were shown to reduce ACE2 expression and subsequently inhibit SARS‐CoV‐2 entry and replication. Therefore, targeting circadian clock genes or their rhythmicity may provide indirect/adjuvant treatment approaches to control ACE2 expression, especially in patients with cardiovascular or respiratory comorbidities. Interestingly, other studies have looked at the regulation of circadian clock rhythmicity with Ang II administration. For instance, Herichova et al. reported that the ACE/ACE2 mRNA ratio showed a clear daily rhythm in the aorta of rats, and that subcutaneous infusion of Ang II modulated the expression of circadian clock genes period circadian regulator 2 (*Per2*) and neuronal PAS domain protein 2 (*Npas2*) which diminished the daily rhythm in ACE/ACE2 mRNA ratio.^112^ Another study conducted by the same research team also showed significant changes in *Per2, Rev‐erbα* and clock‐controlled gene albumin D‐box binding protein (*Dbp*) expression in the heart of rats after Ang II infusion.^113^ Nonaka et al. observed a clear circadian rhythm in *Per2*, *Dbp* and *Bmal1* expression of the mice aorta and showed that treatment with Ang II resulted in a robust upregulation of *Per2* gene expression, followed by a marked downregulation that was subsequently followed by synchronous cycling of *Per2*, *Dbp* and *Bmal1* mRNAs.^114^ Taken together these studies strongly suggest direct and indirect links between ACE/ACE2 expression in epithelial cells and circadian clock gene expression (Figure 3). Additional studies are needed to fully comprehend the direct and indirect links between RAAS and the circadian clock in COVID‐19 infection.

#### 3.3.2 Circadian clock, COVID‐19 and microbiota

The majority of gut microorganisms show a 24‐h oscillatory behaviour that is modulated by the time of the day and food intake. The composition and abundance of the microbial community varies in a time‐dependent manner.^115^ Generally, it is now well accepted that circadian rhythms and gut microbiota are interconnected in a bidirectional manner.^115^, ^116^ Indeed, microbial oscillatory behaviours can be disturbed by the dysregulation of the microbiota host circadian rhythms. Dysregulation of the microbiota‐derived signalling can influence the host circadian clock and affect the circadian gene expression in several tissues, including the immune system, which may impact COVID‐19 disease progression directly or indirectly.^117^, ^118^ Dysbiosis in the host gut microbiome (similar to what is observed in COVID‐19) can be integrated into the host circadian rhythm via several pathways, including changes in microbial pattern‐recognition receptor (PRR) signalling.^117^, ^118^ For example, it was found that changes in PRR signalling due to gut dysbiosis may affect the circadian expression of clock genes in the intestine through myeloid differentiation factor 88 (MyD88)‐induced release of IL‐22 by the innate lymphoid cells in the gut. IL‐22 release leads to the activation of signal transducer and activator of transcription 3 signalling that directly impacts the circadian pattern of gene expression through the core clock genes *Rev‐erbα* and nuclear factor interleukin 3‐regulated protein (*Nfil3*).^119^, ^120^ Moreover, gut dysbiosis due to viral infections can affect the diurnal release and absorption of microbiome‐derived metabolites such as butyric acid which consequently affect the circadian metabolic activity of peripheral organs, including the immune system.^80^ Several other microbiome‐derived metabolites may also influence the circadian activation of AhR.^117^ As mentioned before, it has been suggested that AhR is deeply involved in the immune response to COVID‐19 based on its well‐known role in modulating the antiviral immune response of several other viruses. This includes the murine hepatitis virus which also belongs to the coronaviruses family. Furthermore, it has been shown that the microbiome may influence the circadian clock via epigenetic modifications through the action of microbial histone deacetylase HDAC3.^121^ Finally, recent reports have shown that the microbiome greatly influence the diurnal release of several hormones, including growth and sex hormones.^122^ In fact, clear sex differences in the circadian behaviour of the microbiome can be observed that correspond to the sex differences noted in the circadian oscillations of the host transcriptome. Indeed, Weger et al. showed that that the sex differences in the host hepatic circadian clock are much less pronounced in germ‐free mice and Liang et al. showed that the deletion of *Bmal1* altered the faecal microbiotal configuration in a sex‐dependent way.^123^, ^124^

Despite the aforementioned findings, the interactions between microbiota and viruses are still unclear. It is believed that the microbiota could exhibit two contrasting effects: protecting the body against viral infections via triggering the host immune response (e.g. the protective effect of bacterial flagellin against rotavirus) and evading host antiviral immunity through direct and indirect mechanisms.^125^ Gut dysbiosis has been reported in immunocompromised patients, the elderly, and patients with comorbidities, including cardiovascular disorders, cancers, and autoimmune diseases. Based on the previous factors, it is becoming more evident that the microbiome and the circadian clock are strongly connected. Therefore, investigating the links between SARS‐CoV‐2 infection, circadian clock and gut dysbiosis can provide novel insights into COVID‐19 disease progression, outcomes and response to therapy.^118^

##### Microbiota and COVID‐19 pathophysiology

Gut microbiota‐related studies indicate constitutive ACE2 expression on the luminal surface of small intestinal epithelial cells and colonic crypt cells that shows a non‐RAAS‐related function in modulating neutral amino acid uptake such as tryptophan, mediating intestinal microbiota composition and diversity.^126^, ^127^, ^128^ Hashimoto et al. investigated the role of ACE2 in gut microbial ecology and amino acid uptake using ACE2‐knockout mice and identified reduced levels of neutral amino acids in the serum (e.g. tryptophan).^129^ The uptake of tryptophan was impaired, and the expression of antimicrobial peptides was decreased.^129^ Collectively, these results indicate that amino acid malnutrition, particularly tryptophan, can lead to microbial dysbiosis and can inhibit melatonin synthesis.^129^ Studies in hospitalized COVID‐19 patients have shown gut microbial dysbiosis consisting of a reduction in probiotic bacteria, increased opportunistic pathogens and reduced beneficial symbionts that persisted even after resolution of respiratory symptoms and predicted infection severity.^130^, ^131^, ^132^ Furthermore, studies suggest that a bi‐directional cross‐talk between the gut and lung inflammatory response in COVID‐19 infection may exist, and that their respective microbial composition can be affected by several elements such as lifestyle factors (e.g. diet, smoking and medications).^133^

##### Oral and nasal microbiota, circadian clock and COVID‐19

The oral cavity is the one of the most common entry points for COVID‐19 infection due to the high expression of ACE2, TMPRSS2, sialic acid receptor and CD147, and thus the presence of oral microbiota can play a critical role in this process.^101^ Generally, ACE2 is expressed in more than half of all oral epithelial cells with the highest rate of expression observed on the tongue dorsal surface.^100^, ^134^, ^135^ More precisely, it was found that ACE2 was expressed throughout the layers of stratified squamous epithelium in the tongue and gingivae, whereas TMPRSS2 was mainly localized in the stratum corneum of the tongue along with its salivary coating.^136^ On the other hand, the protease furin was mainly expressed by the basal layers of lingual epithelium or free in the saliva.^136^ The expression of all the aforementioned molecules was also observed in taste‐bud cells in vitro.^136^ The oral cavity ACE2 receptors and microbiota, together or independently, could be promising targets for COVID‐19 treatment or inhibition of infection and spread.^101^ Although various oral microorganisms can translocate to the intestinal microbiota, only some of them are capable of colonizing the gut.^137^ As the oral cavity is considered one of the main entry points for SARS‐CoV‐2, oral microbiota may play a role in a person's susceptibility to the virus infection. To the best of our knowledge, little is known about the link between oral microbiota and the severity of COVID‐19 infection.^138^ One study, however, found that dysbiosis of the oral microbiome was associated with prolonged COVID‐19 symptoms and the development of long‐COVID.^139^ In addition, microbial sequencing studies of the bronchoalveolar fluid of COVID‐19 patients revealed that several oral opportunistic pathogens such as *Capnocytophaga* and *Veillonella* were found in the lungs of COVID‐19 patients, suggesting that the oral cavity may be the source of the several lung co‐infections observed in COVID‐19 patients.^134^ Several risk factors are thought to facilitate the entrance of oral pathogens into the lower respiratory tract through the oral‐lung axis. These include poor oral hygiene, persistent cough and mechanical ventilation.^140^, ^141^ Previous studies indicated that poor oral hygiene leading to oral microbial dysbiosis could accelerate lung injury and deterioration.^141^

Moreover, the damaging effects of the SARS‐CoV‐2 virus on the lung (i.e. inducing hypoxia) may facilitate the growth of these anaerobic pathogens.^134^ In addition, COVID‐19‐mediated oral dysbiosis may exacerbate the disease through the oral‐gut axis as the oral bacteria could alter the gut microbiota, potentially inducing systemic inflammation. For instance, it was found that several oral bacteria such as Fusobacteriaceae and Veillonellaceae were dysregulated in the gut of individuals with inflammatory bowel disease.^142^ Furthermore, it has been reported that other opportunistic oral pathogens (such as *Klebsiella* species) can induce inflammatory colitis in vivo.^143^ Moreover, alterations of the oral microbiota are considered predisposing risk factors for many diseases, including type 2 diabetes, atherosclerotic vascular disease and non‐alcoholic fatty liver disease, which can be linked to periodontal disease, an inflammatory condition of the periodontium associated with oral microbiota dysbiosis.^144^, ^145^ The underlying mechanism for the loss of taste, a common early symptom in COVID‐19 infection, is not fully understood. Oral dysbiosis can increase susceptibility to SARS‐CoV‐2 infection, and ACE2 is expressed in the oral cavity which may be colonized early in COVID‐19 infection. Therefore, it is possible that the loss of taste is due to COVID‐19 disease progression in the oral cavity which can be mitigated by normalizing the oral microbiota. This relationship requires further studies to elucidate the proposed mechanism.^141^, ^146^ Carrouel et al. suggested that some specific mouth rinses may help reduce the SARS‐CoV‐2 viral load in the oral cavity; however, this promising finding should be validated in future studies.^138^

In the context of circadian rhythm, emerging pilot studies have investigated the circadian behaviour of the oral microbiome and its relationship to the biological clock. It was reported that, similar to plasma, 15% of salivary metabolites are regulated by the host internal circadian clock, and that these oscillations persist independently of external circadian cues.^147^, ^148^ In a study investigating the salivary microbiome of six healthy adults for 3 days, Takayasu et al. showed that the metabolic activity of most oral microbes exhibits a clear diurnal rhythm that corresponds to the aerobicity of the microbes, with high aerobic microbial activity observed at noon. Interestingly, the circadian oscillations of the salivary microbiome were almost completely abolished after culturing the saliva in vitro, strongly suggesting that the circadian patterns of oral microbiota are dependent on the host circadian environment.^149^ Collado et al. performed a randomized, crossover study in 10 healthy normal‐weight young women to test the effect of food intake timing on the human microbiota in the saliva and found that food intake timing might affect the diversity and population of oral microbiota.^150^ More interestingly, a very recent study by Sarkar et al. explored the association between the salivary microbiome and concentration of salivary cytokines in 12 healthy adults throughout a 24‐h period. Their analysis identified clear circadian patterns between IL‐1β and *Prevotella*, and IL‐6 with *Prevotella*, *Neisseria* and *Porphyromonas*.^151^ These results strongly suggest that periodic fluctuations of the oral microbiome may regulate the diurnal production of cytokines and the potency of the oral immune response, which may explain how the timing of infection plays a vital role in determining the susceptibility to infections transmitted through the oral cavity.

**FIGURE 4:**
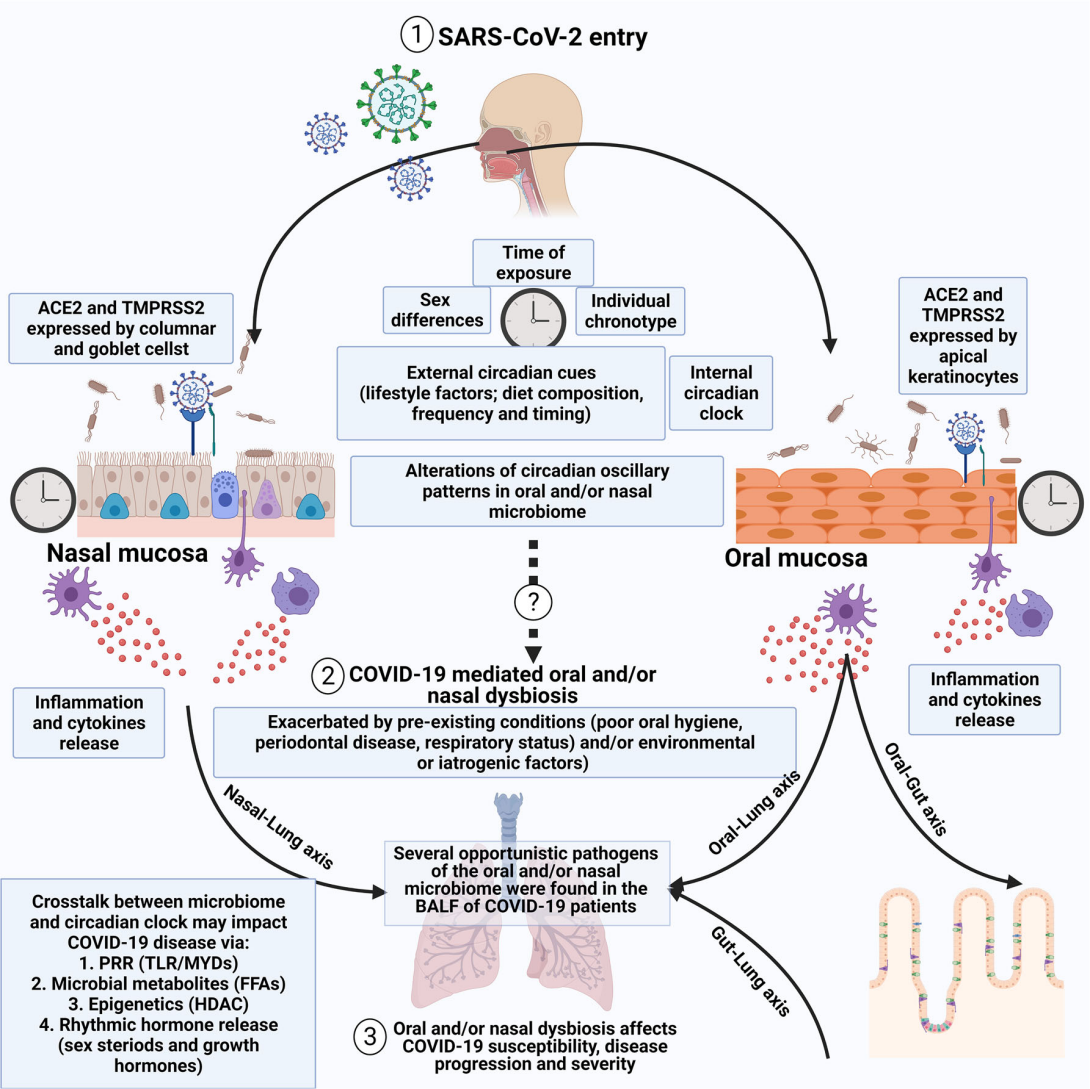
Circadian clock, coronavirus disease 2019 (COVID‐19) and the microbiome. (1) Severe acute respiratory syndrome coronavirus 2 (SARS‐CoV‐2) entry may happen through the oral and nasal cavities. The oral microbiota has been shown to exhibit a strong circadian behaviour influenced by multiple internal and external circadian factors, whereas the circadian behaviour of the nasal microbiome is still understudied. It has been reported that the metabolic activity of the majority of oral microbes exhibits a clear diurnal rhythm that corresponds to the aerobicity of the microbes. Thus, different exposure times to the virus may result in different susceptibilities to viral entry as the microbiotal profile changes during the day. (2) The viral entry may cause oral and/or microbiotal dysbiosis. Several other risk factors are thought to facilitate oral pathogens’ entrance into the lower respiratory tree through the anatomical oral/nasal/lung axis such as poor oral hygiene, periodontal disease, respiratory health status, environmental and/or iatrogenic factors. Although little is known about the link between oral microbiota and the severity of COVID‐19 infection, microbial sequencing studies of the bronchoalveolar fluid (BALF) of COVID‐19 patients revealed that several oral opportunistic pathogens such as *Capnocytophaga* and *Veillonella* were found in the lung of COVID‐19 patients, suggesting that the oral cavity could be the source of the lung co‐infections observed in COVID‐19 patients. It has also been suggested that the bidirectional interactions between gut microbiota and the respiratory mucosa through the physiological gut–lung axis may be involved in regulating the immune responses to SARS‐CoV‐2, which may involve the circadian clock. Indeed, changes in the host microbiome can be integrated into the host circadian via several pathways, including changes in microbial pattern‐recognition receptors (PRR) signalling. Gut dysbiosis due to viral infections can affect the diurnal release and absorption of microbiome‐derived metabolites, including free fatty acids that will consequently affect the circadian metabolic activity of peripheral organs, including the immune system. Furthermore, it has been shown that the microbiome may influence the circadian clock via epigenetic modification through the action of microbial histone deacetylase. Finally, the microbiome greatly affects the diurnal release of several hormones, including growth hormones and sex hormones. (3) Oral and/or nasal dysbiosis may affect COVID‐19 susceptibility, severity and outcomes. ACE‐2, angiotensin converting enzyme 2; FFA, free fatty acids; HDAC, histone deacetylase; TLR, toll‐like receptor; TMPRSS2, transmembrane protease serine 2. *Source*: BioRender.com

Another common entry for SARS‐CoV‐2 infection is the nasal cavity due to the expression of ACE2 and TMPRSS2 by non‐neuronal cells in the olfactory epithelium and olfactory bulb.^101^, ^152^ Indeed, although ACE2 expression is generally low in the respiratory tract, nasal ciliated columnar cells and nasal goblet cells show the highest expression of ACE2 throughout the respiratory tract.^153^ As for nasal microbiota, a sequencing study of the nasal microbiome of COVID‐19 patients by Moore et al. showed that the nasopharyngeal bacterial population changes with the duration of infection with certain bacteria.^154^ For example, *Fusobacterium periodontium*, *Actinomyces* and *Streptococcus* decreased 3 days after infection, whereas other bacteria did not, strongly suggesting the occurrence of nasal microbiotal dysbiosis.^154^ Moreover, it was suggested that CNS involvement via the olfactory neurons may contribute to the smell dysfunction in patients infected with COVID‐19.^101^ Unfortunately, to the best of our knowledge, no comprehensive review hitherto exists in the literature that examined the circadian behaviour of the nasal and respiratory microbiome. Additional studies are necessary to elucidate the links between the oral and nasal microbiome, RAAS system components, circadian clock dysregulation and COVID‐19. SARS‐CoV‐2 entry through the oral and nasal cavity, the involvement of the oro‐nasal microbiome and its relationship to the circadian clock are summarized in Figure 4.

### 3.4 Circadian clock and COVID‐19 disease individual heterogeneity

#### 3.4.1 Demographic determinants

Studies have shown interesting sex differences related to COVID‐19 infection. Females seem to be more resistant to various viral infections (i.e. picornaviruses and hantaviruses) by triggering a more robust immune response than males, probably due to sex‐related differences in cytokine responses to infection.^155^ In fact, Jin et al. compared the disease severity and mortality rate between male and female patients with COVID‐19 infection by extracting data from a case series and a public dataset (Chinese Public Health Science Data Center) and found that COVID‐19 infection in the case series was more severe in men compared to women, and that the number of deceased male patients recorded in the public dataset was 2.4 times higher than female patients.^156^ ‘The immunocompetence handicap model’ is a popular hypothesis for sex‐related outcomes in infectious diseases which suggests that testosterone might be an immunosuppressive factor in males, although results are inconclusive.^157^, ^158^ The anti‐inflammatory role of androgens, and the dual pro‐inflammatory and anti‐inflammatory effects of oestrogen, can help explain the differential impact that sex hormones can have on innate and adaptive immune responses.^157^ ^,^^158^ Moreover, several sex differences have been observed between male and female central and peripheral clocks. Generally, females have a significantly shorter intrinsic circadian period.^159^ Women are more likely to report subjective morning preference than men as they tend to sleep and wake earlier than men.^160^ Using post‐mortem brain tissues, Lim et al. showed that the timing of *PER2*, *PER3* and *BMAL11* expression was relatively advanced in women compared to men, with an estimated phase difference of 4–6 h.^161^ Moreover, electron microscopic analysis indicated that the SCN of male rats has a larger volume with higher numbers of synapses, as compared to females.^162^ Peripherally, Gómez‐Abellán et al. showed that the gene expression of *PER2*, *BMAL1* and *CRY1* was significantly higher in the adipose tissue of women compared to men.^163^ Therefore, considering the circadian clock's role in immunomodulation, sex‐based differences in the circadian behaviour may contribute to the broad varying susceptibility to infections noted between men and women.

#### 3.4.2 Genetic determinants

As ACE2 is one of the most critical host receptors for SARS‐CoV, the specificities of interaction between the variants of ACE2 and the spike protein of SARS‐CoV are major determinants of host cell tropism. The differences noted in the susceptibility to infection, symptoms and outcomes in COVID‐19‐infected patients can be due to different expression levels and expression profiles of human ACE2 in various tissues.^164^, ^165^

Population‐based genetic analysis using different tissues (adipose visceral omentum, skeletal muscle, mammary tissue, testis, subcutaneous adipose, prostate, tibial nerve and artery, and pituitary and brain) found that East Asian populations had higher allele frequencies in expression quantitative trait loci variants associated with increased ACE2 expression compared to Admixed American, African, European and South Asian populations, suggesting differential response to COVID‐19.^165^ There is evidence of poorer clinical outcomes among Asians in population‐based studies. A study using de‐identified hospital discharge data from the USA from March 2020 to September 2020 (*n* = 181 813 hospitalized adult patients with COVID‐19) found that individuals from Asian and Hispanic/Latino ethnicities had a higher risk of intensive care unit (ICU) admission, invasive mechanical ventilation and death compared to White patients when adjusting for age, sex, insurance type, discharge month, US Census region, anaemia, heart disease, diabetes, obesity, renal failure and coagulopathy.^26^ Furthermore, a meta‐analysis pooling data from 50 studies in the USA and the United Kingdom found that individuals of Asian ethnicities were at a higher risk of COVID‐19 infection, increased ICU admission and mortality; however, there are certain inconclusive results based on study design (inpatient and outpatient, hospitalized patients only or general population, peer‐reviewed literature, etc.).^166^ Interestingly, studies have indicated a disproportionately high prevalence of COVID‐19 hospitalizations among individuals of Black ethnicities,^166^, ^167^ although recent evidence has concluded that there is not a significant difference in COVID‐19‐related mortality when comparing these groups.^166^ The results suggest that susceptibility to COVID‐19 is not solely a genetic predisposition; rather, there is an interplay with environmental or other factors influencing clinical outcomes. The lack of consensus on the ethnocentric risk of COVID‐19 highlights the need for a comprehensive approach incorporating unique characteristics of each individual such as age, sex, nutritional, socioeconomic, general health (various comorbidities, including metabolic, autoimmune, cardiovascular, respiratory diseases, cancer, etc.), circadian clock status, geographic location, to improve COVID‐19 clinical outcomes.

It has been shown that the distribution of *PER2* polymorphisms might differ in people living in different geographical locations.^168^ Cruciani et al. genotyped *PER2* in 499 unrelated individuals from Africa (*n* = 131), Europe (*n* = 154), Asia (*n* = 136) and Native Americans (*n* = 78) and, although they found no evidence of latitude's effect on *PER2* variability, they noticed that there was a statistically significant difference in geographic distribution of *PER2* polymorphisms by continent, and that this diversity was even more pronounced when populations were grouped as African and non‐African. Thus, they concluded that positive selection and genetic drift might have influenced the continental differences in *PER2* polymorphisms.^168^

Still, the relationship between how genetic traits and different circadian clock profiles can influence the susceptibility and outcomes of COVID‐19 infection remained largely unexplored and has potential therapeutic benefit.

#### 3.4.3 Epigenetic determinants

Given the heterogeneous nature of the symptomology of COVID‐19, it is now widely accepted that its pathophysiology may also involve epigenetic factors. Recent reports support the hypothesis that coronaviruses regulate the host epigenome through elaborated and well‐controlled process interfering with the host innate immune antiviral defence mechanisms, thereby promoting robust viral replication and pathogenesis.^169^, ^170^ Small non‐coding RNAs, termed microRNAs, are known to regulate gene expression at the posttranslational and/or post‐transcriptional level by targeting mRNAs. A recent study by Nepotchatykh et al. identified microRNAs targeting key regulators of the circadian rhythm like SIRT1 (hsa‐miR‐291‐3p and hsa‐miR‐181a‐5p), PER2 (hsa‐miR‐28‐5p) and PER3 (hsa‐miR‐29a‐3p and has‐miR‐181b‐5p) in the context of myalgic encephalomyelitis/chronic fatigue syndrome (ME/CFS).^171^ The biological and clinical relevance of this condition with COVID‐19 stem from the fact that over 75% of persons with ME/CFS described episodes of viral infection as a trigger, which includes coronavirus.^172^, ^173^, ^174^ Of note, melatonin and phytomelatonin, a plant derivative also known as *N*‐acetyl‐5‐methoxytryptamine, can increase the expression of hsa‐miR‐29a‐3p.^175^ Another study reported a significant downregulation of hsa‐miR‐181b‐5p in cells treated with Ang II.^176^ It remains to be demonstrated if the modulation of these circulating microRNAs through melatonin supplement or melatonergic drugs could attenuate SARS‐CoV‐2 viral infection by boosting/resynchronize the immune system through key circadian regulators like PER2 and PER3 genes.

#### 3.4.4 Unique determinants of individual circadian profiles

One of the critical factors determining the morbidity and mortality of COVID‐19 infection is the response of the adaptive immune system to SARS‐CoV‐2 which plays a vital role in the management of the viral load.^177^ SARS‐CoV‐2 infection activates TLR7 followed by the production and secretion of TNF‐α, IFN‐α, IL‐12 and IL‐6, activation of virus‐specific cytotoxic CD8^+^ T cells, the differentiation of antigen‐specific B cells and the production of antibodies. A dysregulated innate immune response and inadequate development of an effective adaptive immune response, common to populations at risk (people with comorbidities and the elderly), can lead to persistent self‐induced inflammation and even death.^177^

The current knowledge of the immune system and the circadian clock suggests that our body is more susceptible to some respiratory viruses in the early morning, highlighting a need to determine if our immune system is more vulnerable to viral pathogens at certain times of the day. This information could help control the COVID‐19 pandemic (and possible future pandemics) by exploiting the individual circadian clock profile and susceptibility to a given infection/disease to define the most appropriate working and/or social distancing schedules that would diminish the chance of various infections, particularly for the higher risk groups.^5^

#### 3.4.5 Individual health status and comorbidities

Yang et al. pooled data for a meta‐analysis from 7 studies with 1576 laboratory‐confirmed cases of COVID‐19 at Chinese hospitals (890 males, 686 females, mean age of 49.6‐year old) to examine the prevalence of comorbidities in a Chinese cohort of patients infected with COVID‐19. They found that patients with hypertension, cardiovascular and respiratory diseases showed severe morbidity.^178^ Many of the patients with severe symptoms of COVID‐19‐related disease were elderly individuals with comorbidities including kidney, liver, cardiovascular diseases or malignant tumours.^25^ Similarly, a large‐scale study with 5700 hospitalized patients (3437 males, 2263 females, median age of 63‐year old) who tested positive for COVID‐19 conducted in the United States showed that the prevalence of SARS‐CoV‐2 infection was higher in patients with hypertension, obesity and/or diabetes. There was a consistent trend of increasing mortality with increasing age among men and women, with the highest fatality rates among those aged 80 years or older. Across all age groups (aged 20 years old or older), men had higher mortality compared to women.^21^

Cai et al. have investigated how obesity, a common chronic disease, can affect the outcome of COVID‐19 infection by analysing the disease progression in a hospitalized Chinese cohort of 383 COVID‐19 patients who were classified into the following four categories based on their body mass indexes: underweight, normal weight, overweight and obese. The risk of developing severe pneumonia in overweight patients was 86% higher than in normal weight patients. The obese patients presented 2.42‐fold higher odds of developing severe pneumonia than patients with normal weight. Taken together, the study concluded that obesity can significantly increase the chance of severe pneumonia in COVID‐19‐infected patients.^179^

The circadian clock directly or indirectly regulates most of comorbidities found in COVID‐19 patients with severe outcomes. For instance, multiple reports showed that the circadian clock plays a vital role in regulating blood pressure, and healthy individuals experience a 10%–20% ‘dip’ in blood pressure at night.^180^, ^181^ Circadian misalignment (related to inversion of the behavioural and light/dark cycle, typical in shift workers) results in an increased incidence of blood pressure disorders, and also several clock genes knock‐out rodent models showed altered BP phenotypes.^182^, ^183^, ^184^, ^185^ Several studies showed that night‐shift workers with circadian misalignment have a higher risk of obesity and diabetes.^186^, ^187^ Generally, the strong influence of the circadian clock on obesity and metabolic disorders is well documented in the literature and has been thoroughly covered in other reviews.^188^, ^189^

#### 3.4.6 Lifestyle and environmental factors

Additional factors that may affect the susceptibility, disease progression, outcome and recovery from SARS‐CoV‐2 infection may also include lifestyle (i.e. smoking, nutrition, irregular behavioural, light/dark cycle and sleep‐wake patterns) and environmental factors (i.e. air pollution, toxin exposure).

There is a lack of consensus in the current literature on the role of cigarette smoking in the upregulation or downregulation of ACE2 expression in the lungs,^190^, ^191^, ^192^, ^193^, ^194^ rate of SARS‐CoV‐2 infection^195^, ^196^, ^197^ and severity of symptoms,^196^, ^197^, ^198^ warranting further studies. Leung et al. investigated whether patients with chronic obstructive pulmonary disease (COPD) have an upregulated expression of ACE2 in lung tissue specimens from 10 current smokers with COPD, 9 non‐smoker healthy controls and 8 healthy current smokers. They found ACE2 expression in the lungs was higher in current smokers compared to never smokers and those with COPD, compared to patients without COPD. Furthermore, they suggested a dose–response relationship and increased ACE2 expression in current smokers compared to former smokers.^190^ Similarly, ACE2 expression in human lung tissue was found to have a dose–response and a 30%–55% increased expression when comparing cigarette smokers with non‐smokers, and a 40% decreased expression in former smokers compared to current smokers.^191^ These findings are consistent with results from a study by Brake et al. which found upregulated ACE2 expression when comparing COPD patients to healthy controls, and cigarette smokers to never smokers.^192^ These studies suggest cigarette smoking may increase the risk of coronavirus infections in active smokers and those with COPD through the upregulation of ACE2 in lung tissues. However, there is contradictory evidence albeit using rat models. Two studies concluded that cigarette exposure decreased ACE2 expression in lung sections of rats when compared to the non‐exposed group.^193^, ^194^ Although at face value these findings may seem contradictory, it is possible that the increased ACE2 expression may be an adaptive response over time due to chronic cigarette exposure^191^ that may not be apparent in the rat models due to potential heterogeneity related to the type of organism, the dose and duration of exposure. Due to the limitations in study design, data collected (current/former/never smokers, number of patients sampled etc.), geographical variation, the role of cigarette smoking and COVID‐19 infection and severity, the relationship between smoking and COVID‐19 severity requires further research. However, individuals with comorbidities related to cigarette smoking (e.g. COPD, cardiovascular disease, diabetes) have been identified as having worse COVID‐19 disease outcomes.

Several studies indicated that smoking can disrupt circadian clock functions.^199^ Casale et al. showed that passive smoking changed the circadian rhythm of peak respiratory flow in children.^200^ Hwang et al. studied the effect of environmental tobacco/cigarette smoke on the circadian clock rhythms in C57BL/6J and *Bmal1*‐floxed mutant mice models cross‐bred with Clara cells 10‐kDa cre (*CC10*‐cre) to conditionally delete *Bmal1* in the lung tissues. They found that cigarette smoke can disrupt the circadian clock expression in lung and brain tissues, increasing lung inflammation and emphysema. This study also collected lung tissue specimens from non‐smokers, smokers and patients with COPD and found that BMAL1 was downregulated in the patients with COPD. The downregulation of BMAL1 was mechanistically linked to the SIRT1‐BMAL1 pathway and associated with abnormal lung inflammation.^199^

Irregular behavioural, light/dark cycle and wake/sleep pattern can lead to negative health consequences, including a weakened immune system which can be attributed to circadian disruption.^201^, ^202^ A recent study found shift working healthcare workers had an increased risk of influenza‐like illness and/or acute respiratory illness when compared to non‐shift working healthcare workers, with the authors concluding that shift work increases susceptibility to infectious diseases likely through immunological pathways.^203^ Another study examining the effects of night‐shift work on healthcare professionals found that chronic exposure to night‐shift work as well as recent night‐shift work may influence the immune state (higher levels of monocytes and B and T lymphocytes).^204^ Disturbed expression of circadian clock proteins such as BMAL1 may partially explain this finding as they regulate the level of monocytes.^204^ In BMAL1 knockout mice, the loss of the diurnal variation in the recruitment of inflammatory monocytes was shown to predispose mice to the development of pathologies associated with acute and chronic inflammation.^205^ In relation to COVID‐19 infection and hospitalization, a population‐based study conducted by Fatima et al. to examine the effects of shift work on the incidence of COVID‐19 infection using the UK Biobank cohort data from March to September 2020 concluded that shift workers, specifically night‐shift workers, were at an increased risk of COVID‐19 infection.^206^ This may be due to circadian disruption leading to poorer immunological outcomes, possibly through reduced melatonin levels.^206^

Evidence suggests that air pollution can worsen the CFR in COVID‐19‐infected patients. Yao et al. found a positive association between fine particulate matter (PM) pollution smaller than 2.5 (PM_2.5_) or 10 μm (PM_10_) and the CFR in Wuhan, China.^207^ Zhu et al. studied the association between SARS‐CoV‐2 infection and air pollution in China by analysing the relationship between six air pollutants, including PM_2.5_, PM_10_, SO_2_, O_3_, NO_2_ and CO, and newly diagnosed COVID‐19 cases over a 2‐week period. They found that the amount of PM_2.5_, PM_10_, O_3_ and NO_2_ was positively associated with the number of newly confirmed COVID‐19 cases.^208^ The relationship between air pollution and the circadian clock remains unclear. However, air pollution induces oxidative stress which has direct circadian toxic effects as several core clock proteins are sensitive to redox changes. Indeed, Haberzettl et al. have proposed that air pollution will disturb the lung peripheral clock by inducing oxidative stress in the pulmonary tissues.^209^ Based on the previous description, exposure to air pollution may disrupt the circadian clock, potentially complicating the COVID‐19 outcome.

### 3.5 Circadian clock – an emerging therapeutic target to improve COVID‐19 outcomes

#### 3.5.1 Manipulating the clock with lifestyle changes

##### Diet and COVID‐19

Diet and nutrition are vital for a healthy immune system. It is known that some micronutrients can have a critical impact on the modulation of immunity.^210^ These micronutrients, such as vitamins A, B6, B9, B12, C, D, E, iron, selenium, copper, zinc and magnesium, can support host resistance to viral infections, including SARS‐CoV‐2.^210^

Studies have suggested that Western diet or a diet consisting of high saturated fat can harm the adaptive immune system by increasing oxidative stress, leading to the inhibition of T and B lymphocyte functions.^211^ Horne and Vohl looked for a potential connection between ACE2 expression, dietary fat, resveratrol (a polyphenolic compound found in berries and peanuts) and the severity of COVID‐19 infection by reviewing the very few existing studies on this matter and concluded that lower dietary fat and/or higher resveratrol intake might impact the body's immune response to SARS‐CoV‐2. It is worth mentioning that patients with DM tend to have a high‐fat diet (HFD), which might explain in part why COVID‐19 symptoms are more severe in patients with DM and people with hypertension, which frequently occur together.^212^, ^213^, ^214^

A systematic and meta‐analysis study conducted on 25 eligible randomized controlled trials (total of 11 321 participants, aged 0–95 years) showed the preventive effect of vitamin D on acute respiratory tract infection,^215^ which suggests that vitamin D supplementation might increase resistance to SARS‐CoV‐2.^216^ In addition, vitamin D supplementation was shown to significantly reduce serum inflammatory markers associated with COVID‐19.^217^

##### Diet and the circadian clock

Hsieh et al. investigated the relationship between obesity linked to an HFD and expression of the circadian clock and clock‐controlled genes in peripheral tissues in male obese mice. Their findings showed alterations in the expression of *Bmal1*, *Per1‐3*, *Cry 1–2*, *Ck1ɛ*, *Nhe3*, *Pdk4*, *Pepck*, *Dbp* and *E4bp4* in the liver and/or kidneys directly linked to an HFD.^218^ Kohsaka et al. explored whether circadian clock rhythms are associated with obesity and diabetes by recording the locomotor activity in two groups: one fed regular chow and another an HFD.^219^ They observed an increase in the length of the circadian period only in the HFD‐fed mouse group.^219^ It is interesting to note that the study found no association between period change and body weight change in this group during the 6‐week duration of the study. The authors also observed a severe attenuation in the amplitude of *Clock* rhythm in fat tissue and less attenuation in the liver of mice fed an HFD. In addition, *Per2* was downregulated and the amplitude of *Bmal1* expression was decreased in liver and fat tissue.^219^ These results demonstrate a clear link between an HFD and alterations of clock gene expression at both tissue‐ and gene‐specific levels.^219^

Interestingly, Hong et al. have shown that an HFD can trigger the NF‐κB signalling pathway in mice, leading to the modulation of circadian clock core genes and circadian locomotor behaviour. Their study suggested a cross‐regulatory link between inflammation, HFD and the circadian clock.^220^, ^221^ However, the relationship between circadian clock machinery, diet and COVID‐19 has not yet been thoroughly investigated.

#### 3.5.2 Manipulating the clock with pharmacological interventions

Ang II impacts the circadian system by affecting melatonin production, leading to the modulation of the circadian system. This is due to modulation of the synthesis and activity of the tryptophan hydroxylase enzyme by Ang II, which limits melatonin production.^222^ A study‐analysed melatonin's effect on a podocyte‐injury in an in vitro model and found that melatonin reduced Ang II‐induced apoptosis and increased cell proliferation via the downregulation of the production of apoptotic proteins such as caspase‐3 and BAX and a change in the BAX/BCL‐2 ratio.^223^ Ishigaki et al. studied the relationship between chronic kidney disease (CKD) and hypertension in 53 patients and found that impaired melatonin secretion at night may be linked with night‐time intrarenal RAAS activation, followed by renal damage in CKD patients.^224^

Melatonin has anti‐inflammatory and antioxidant characteristics that can protect the body against acute lung injury/ARDS caused by viruses and pathogens.^59^ Reduction in vascular permeability, sedative effects, anti‐anxiety effects and improved sleep quality are other benefits of melatonin which may improve the quality of life and clinical outcomes of COVID‐19 patients.^59^ Indeed, Zhang et al. recommended melatonin for its anti‐inflammatory and antioxidant benefits as a potential adjuvant therapy for COVID‐19.^59^ Shneider et al. suggested melatonin can reduce the severity of symptoms in COVID‐19‐infected patients and help them recover after the active phase of infection has ended.^225^ A clinical trial aimed to identify the efficacy and doses of melatonin to fight against SARS‐CoV‐2 reported that the intravenous administration of melatonin to ICU patients suffering from SARS‐CoV‐2 infection has been approved by the Spanish Agency of Medicines and Medical Devices.^226^

It seems plausible to assume that patients infected with SARS‐CoV‐2 may show sleep disturbance symptoms as Ang II can affect melatonin production. It is known that higher levels of Ang II are associated with obstructive sleep apnoea that can also cause hypertension through stimulation of RAAS.^227^ Carhan et al. showed that loss of ACER (an ACE2 homolog in *Drosophila melanogaster*) could disrupt the night‐time sleep.^228^ To our knowledge, no study has investigated the prevalence of COVID‐19 infection in patients who have pre‐existing sleep disorders. Vitale et al. monitored four COVID‐19 patients’ sleep quality during their sub‐acute recovery stage using wrist actigraphy and found that sleep efficiency and immobility time were lower in the patients who experienced the most severe respiratory symptoms compared to those who experienced mild respiratory symptoms without a need for ICU stay.^229^

#### 3.5.3 Manipulating the clock and COVID‐19 recovery

Chronotherapy is a promising approach which involves tailoring treatment to an individual based on their circadian profile to improve the effectiveness of the therapeutic and reduce adverse effects.^230^ The pharmacokinetics and pharmacodynamics of different drugs are known to change over 24‐h time periods influencing their efficacy, with approximately 50% of drugs targeting a gene under circadian control.^231^ In severe cases of COVID‐19 characterized by the cytokine storm, timing the dosage of anti‐inflammatory drugs to coincide with the peak of detrimental inflammatory mediators (afternoon), while avoiding steady‐state levels to allow effective inflammatory response to the virus, may improve COVID‐19 disease outcomes.^232^

Melatonin has indirect anti‐viral actions through anti‐inflammation, anti‐oxidation and immune enhancing characteristics which may potentially mitigate lung injury and other inflammatory consequences of COVID‐19.^59^ It is relatively safe and currently undergoing testing as a possible adjuvant in the treatment of critically ill COVID‐19 patients.^233^ Additionally, other circadian modulators have shown anti‐viral properties such as REV‐ERB agonists which inhibit HCV entry, RNA replication and release of infectious particles in murine models. Furthermore, CRY stabilizers and ROR inverse agonists may potentially modulate inflammatory and viral responses, warranting further research.^234^

Timing the COVID‐19 vaccination with an individual's circadian profile may result in a more effective coordinated host–response. Studies suggest hepatitis A and influenza vaccinations were more effective in the morning than afternoon as measured by a higher antibody titre.^235^, ^236^ Therefore, based on the immunomodulatory effects of the circadian clock, and with the global roll‐out of the COVID‐19 vaccines, studies need to account for timing of vaccination to optimize host–response.

## 4 CONCLUSIONS AND FUTURE PERSPECTIVES

**FIGURE 5:**
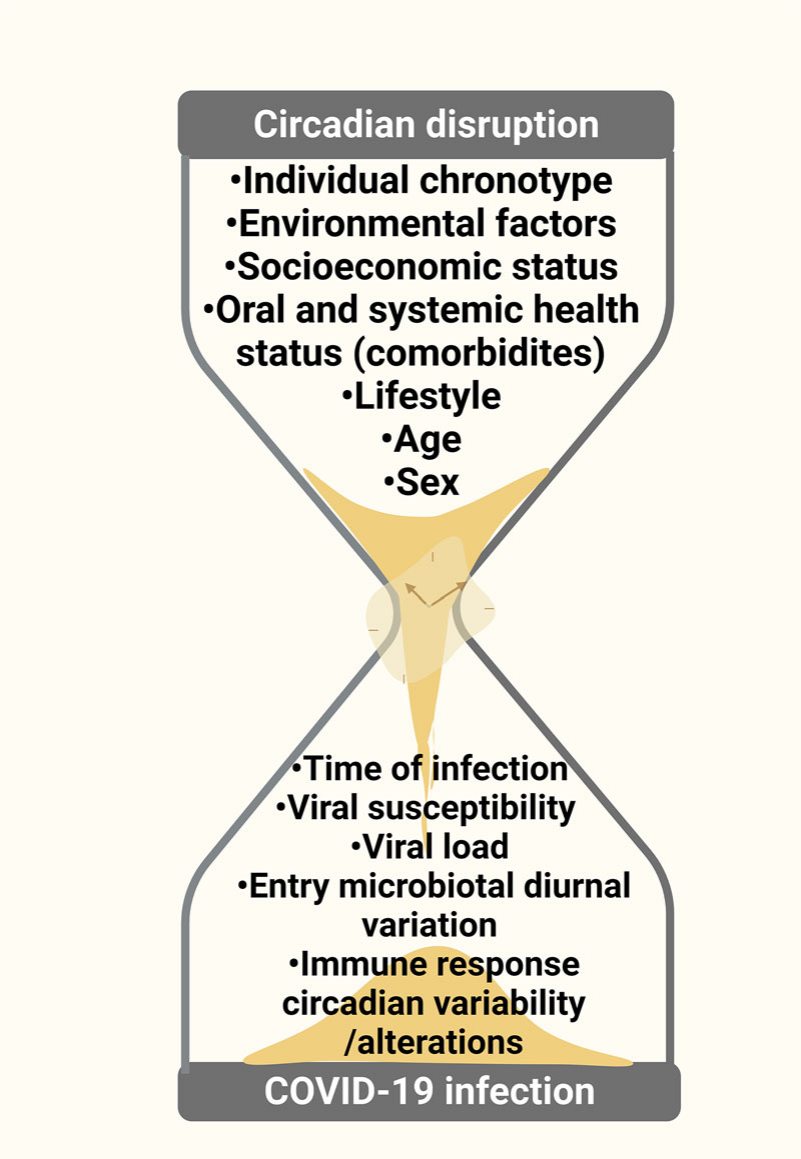
Determinants of the individual circadian clock profile and coronavirus disease 2019 (COVID‐19) disease heterogeneity. Misalignment of the behavioural (fasting/feeding and sleep/wake pattern) and light/dark cycles can lead to circadian disruption; the circadian profile may display a high individual heterogeneity reflective of a combination of various determinants (including age, sex, socioeconomic status), genetic heterogeneity, individual chronotype, oral and systemic health status (e.g. comorbidities, microbiome composition), lifestyle, geographical location and environmental factors and so on that can impact the susceptibility, severity and prognosis of COVID‐19‐related disease. *Source*: BioRender.com

The severity of COVID‐19 outcomes could be impacted by patients’ demographic characteristics, genetic profile, immune system, comorbidities (such as hypertension, respiratory system diseases, cardiovascular diseases, diabetes, obesity) and other factors (including lifestyle and environmental factors), many of which are based on, or related to, the individual's circadian rhythm profile (Figure 5). The role of clock genes and the therapeutic benefits of melatonin in several viral infections have been reported. Based on the evidence summarized here, it is likely that there are many potential connections between circadian rhythms, clock genes and SARS‐CoV‐2 that warrant further investigation. For instance, women seem more resistant to COVID‐19 infection with less severe outcomes, likely due to their shorter intrinsic circadian period and more robust immune response compared to men. People with disrupted circadian rhythms, such as individuals with sleep disorder diseases, diabetes, cardiovascular disorders and chronic oral inflammation, may be at a higher risk of severe outcomes. As a result, novel therapeutic approaches that take into account individual circadian clock profiles may contribute to improving the outcome of COVID‐19‐infected patients. In addition, biomarkers of circadian clock disruption or alterations may be useful complementary tools in predicting clinical outcomes in COVID‐19‐infection.

## CONFLICT OF INTEREST

The authors declare no conflict of interest.
